# The Value of Semiquantitative Parameters Derived from ^18^F-FDG PET/CT for Predicting Response to Neoadjuvant Chemotherapy in a Cohort of Patients with Different Molecular Subtypes of Breast Cancer

**DOI:** 10.3390/cancers14235869

**Published:** 2022-11-29

**Authors:** Luca Urso, Laura Evangelista, Pierpaolo Alongi, Natale Quartuccio, Corrado Cittanti, Ilaria Rambaldi, Naima Ortolan, Francesca Borgia, Alberto Nieri, Licia Uccelli, Alessio Schirone, Stefano Panareo, Gaspare Arnone, Mirco Bartolomei

**Affiliations:** 1 Department of Translational Medicine, University of Ferrara, Via Aldo Moro 8, 44124 Ferrara, Italy; 2 Nuclear Medicine Unit, Oncological Medical and Specialist Department, University Hospital of Ferrara, 44124 Cona, Italy; 3 Department of Medicine DIMED, University of Padua, 35128 Padua, Italy; 4 Nuclear Medicine Unit, ARNAS Ospedali Civico, Di Cristina e Benfratelli, 90127 Palermo, Italy; 5 Nuclear Medicine Unit, Ospedali Riuniti Villa Sofia-Cervello, 90146 Palermo, Italy; 6 Oncology Unit, Oncological Medical and Specialists Department, University Hospital of Ferrara, 44124 Ferrara, Italy; 7 Nuclear Medicine Unit, Oncology and Haematology Department, University Hospital of Modena, 41125 Modena, Italy

**Keywords:** FDG PET/CT, FDG, semiquantitative parameters, SUVmax, MTV, TLG, breast cancer, neoadjuvant chemotherapy, pCR after NAC, survival prediction

## Abstract

**Simple Summary:**

The aim of this study was to investigate whether baseline [^18^F]Fluorodeoxyglucose ([^18^F]FDG) positron emission computed tomography/computed tomography (PET/CT) could predict pathological complete response (pCR) after neoadjuvant chemotherapy (NAC) and survival outcomes in patients affected by different molecular subtypes of breast cancer (BC). Semiquantitative parameters, extracted from baseline [^18^F]FDG PET/CT, seem to be promising in the prediction of response to NAC in Luminal B and Luminal B + HER-2 patients and in the survival prediction of triple negative BC patients achieving pCR after NAC. PET/CT scan with advanced parameter analysis could carve out a synergic role, together with other imaging tools, for a more accurate evaluation of these patients at diagnosis.

**Abstract:**

Pathological complete response (pCR) after neoadjuvant chemotherapy (NAC) is a strong prognostic factor in breast cancer (BC). The aim of this study was to investigate whether semiquantitative parameters derived from baseline [^18^F]Fluorodeoxyglucose ([^18^F]FDG) positron emission computed tomography/computed tomography (PET/CT) could predict pCR after NAC and survival outcomes in patients affected by different molecular subtypes of BC. We retrospectively retrieved patients from the databases of two Italian hospitals (Centre A: University Hospital of Ferrara; Centre B: University of Padua) meeting the following inclusion criteria: (1) diagnosis of BC; (2) history of NAC; (3) baseline [^18^F]FDG PET/CT performed before the first cycle of NAC; (4) available follow-up data (response after NAC and survival information). For each [^18^F]FDG PET/CT scan, semiquantitative parameters (SUVmax, SUVmean, MTV and TLG) related to the primary tumor (B), to the reference lesion for both axillary (N) and distant lymph node (DN), and to the whole-body burden of disease (WB) were evaluated. Patients enrolled were 133: 34 from centre A and 99 from centre B. Patients’ molecular subtypes were: 9 luminal A, 49 luminal B, 33 luminal B + HER-2, 10 HER-2 enriched, and 32 triple negative (TNBC). Luminal A and HER-2 enriched BC patients were excluded from the analysis due to the small sample size. pCR after NAC was achieved in 47 patients (41.2%). [^18^F]FDG PET/CT detected the primary tumor in 98.3% of patients and lymph node metastases were more frequently detected in Luminal B subgroup. Among Luminal B patients, median SUVmean_B values were significantly higher (*p* = 0.027) in responders (7.06 ± 5.9) vs. non-responders (4.4 ± 2.1) to NAC. Luminal B + HER-2 non-responders showed a statistically significantly higher median MTV_B (7.3 ± 4.2 cm^3^ vs. 3.5 ± 2.5 cm^3^; *p* = 0.003) and TLG_B (36.5 ± 24.9 vs. 18.9 ± 17.7; *p* = 0.025) than responders at baseline [^18^F]FDG PET/CT. None of the semiquantitative parameters predicted pCR after NAC in TNBC patients. However, among TNBC patients who achieved pCR after NAC, 4 volumetric parameters (MTV_B, TLG_B, MTV_WB and TLG_WB) were significantly higher in patients dead at follow-up. If confirmed in further studies, these results could open up a widespread use of [^18^F]FDG PET/CT as a baseline predictor of response to NAC in luminal B and luminal B + HER-2 patients and as a prognostic tool in TNBC.

## 1. Introduction

Breast cancer (BC) is the leading cause of cancer-related death among females worldwide and constitutes approximately 15% of all cancer-related deaths in females [1]. BC is a complex neoplasm in terms of management and prognostic assessment due to its multiple clinical and pathological variables [2]. The receptor status (i.e., the expression of estrogen receptor-ER, the progesteron receptor-PR, and the human epidermal growth factor receptor 2—HER-2) is considered essential in the clinical management of BC. Indeed, the definition of luminal categories in BC patients has significantly affected the choice of therapy in any setting of disease, from the diagnosis to the management of metastasis. Luminal A and B (positive ER and negative HER-2) are the most common subtypes, followed by HER-2 enriched and triple negative cancer (TNBC). However, the prognosis of the luminal group is more favorable than the others [3]. Locally advanced breast cancer (LABC) occurs in approximately 30% of patients at the time of diagnosis, and it can be treated with neoadjuvant chemotherapy (NAC) for both testing the chemosensitivity and reducing the size of the tumor [4].

A growing interest around NAC in BC has been reported in recent years, although there are still many variables to settle about this issue (i.e., patients selection, therapeutic agents and number of therapeutic schemes), and a standardized treatment is far from being defined [5]. Based on available literature data, NAC did not demonstrate a clear advantage over conventional adjuvant chemotherapy in terms of outcomes. [6,7]. However, NAC was correlated to BC downgrading, and consequently, to an easier and more conservative breast surgery in a large portion of patients [8]. Moreover, pathological complete response (pCR) after NAC is a recognized strong prognostic factor in BC, particularly in TNBC and HER-2 enriched BC [8]. Breast magnetic resonance imaging (MRI) has provided encouraging results in the assessment of response after NAC, becoming the gold standard modality for pre-surgical treatment response assessment [9]. However, breast MRI can provide only an evaluation of T and N staging after NAC, and several factors can reduce its accuracy, including molecular subtypes (Luminal BC), chemotherapy regimen (taxane and antiangiogenetic drugs), and therapy-induced inflammation and fibrosis [9,10]. Therefore, in recent years [^18^F]Fluorodeoxyglucose ([^18^F]FDG), positron emission tomography/computed tomography (PET/CT) has been evaluated with growing interest in BC patients addressed to NAC, as it can provide an in vivo functional evaluation of the metabolism of a cancer lesion before and after therapy [11,12,13]. The possibility of predicting pCR after NAC through baseline imaging data would be of great clinical meaning in BC patients, determining a more tailored treatment approach.

Based on these premises, the aim of our work was to assess the possible role of volumetric parameters derived from baseline [^18^F]FDG PET/CT to predict response after NAC in a cohort of patients with different molecular subtypes of BC. Moreover, we explored the possible prognostic value of volumetric parameters, correlating them with patients’ outcomes in terms of disease-free survival (DFS) and overall survival (OS).

## 2. Materials and Methods

### 2.1. Patients Selection

We retrospectively analyzed 133 patients referring to two Italian hospitals (Centre A: University Hospital of Ferrara; Centre B: University of Padua) for the management of BC between February 2015 and August 2020. Inclusion criteria were: (a) histologically proven diagnosis of BC; (b) history of NAC; (c) baseline [^18^F]FDG PET/CT scan performed prior to starting NAC; (d) availability of histopathology and follow-up survival data. Patients who did not undergo surgery after NAC were excluded from the study. For every patient, data regarding histology, hormone receptor, and HER-2 status, proliferation rate (expressed by Ki67 index), and BRCA genetic analysis of the BC were retrieved.

The patients were classified according to the St. Gallen consensus [14] into 5 molecular subtypes subgroups: (1) Luminal A (ER-positive and/or Progesterone Receptors (PR)-positive and HER-2 negative; Ki67 < 20%); (2) Luminal B (ER-positive and/or PR-positive and HER-2 negative; Ki67 ≥ 20%); (3) Luminal B + HER-2 (ER-positive and/or PR-positive and HER-2 positive; Ki67 ≥ 20%); (4) HER-2 enriched BC (without hormonal receptors expression and HER-2-positive); (5) TNBC (without expression of both hormonal receptors and HER-2 protein).

Before NAC, a clip was placed to identify the residual disease. NAC regimen was selected on the basis of the histological features of BC, according to the most updated guidelines [15]. The following NAC regimen were used:

- Luminal B BC: Epirubicin and cyclophosphamide (EC) or doxorubicin and cyclophosphamide (AC) for 16 patients; Epirubicin (EPI) + docetaxel for 33 patients.
- Luminal B + HER-2 BC: EC/AC + trastuzumab for 26 patients; EPI + docetaxel + Trastuzumab for 7 patients.
- TNBC: EC/AC for 11 patients; EPI + docetaxel for 21 patients.

After NAC, surgery was performed.

### 2.2. Response Assessment and Follow-Up

The Sataloff criteria [16] were considered for the evaluation of response to NAC. A pCR was defined as the complete absence of residual invasive tumor cells on microscopy both in the breast and in the axillary or distant lymph nodes. All patients with tumors showing progression, stable disease, or a partial response after NAC were classified as non-responders.

To evaluate patients’ survival, follow-up data were retrieved from the hospital archives of both centres. Patients with missing data were contacted with phone interviews. Outcomes were evaluated in terms of DFS, defined as the time (months) from [^18^F]FDG PET/CT to the evidence of disease relapse at imaging or histopathology, and OS, defined as the time (months) from the first cycle of NAC to death from any cause.

### 2.3. Image Acquisition

The patients were required to fast for at least 6–8 h and maintain adequate hydration before the [^18^F]FDG PET/CT scans. Diabetic patients had their blood glucose levels measured before [^18^F]FDG administration. Patients with glucose values above 200 mg/dL were rescheduled. Images were acquired 50–70 min after [^18^F]FDG injection (3.5 MBq/Kg) following a standardized acquisition protocol recommended by EANM guidelines [17] on a dedicated PET/CT scanner (Centre A: Biograph mCT Flow; Siemens Medical Solution, Malvern, PA, USA—Centre B: Biograph 16; Siemens Medical Solution, Hoffman Estates, IL, USA). A concomitant low-dose CT scan (120 kV and 80 mA/s) was performed for the attenuation correction of the PET emission data acquired from the mid-thigh to the skull vertex.

### 2.4. Response Assessment and Follow-Up

In both centres, the PET/CT images were all processed and analyzed using a Syngo.via Workstation (Siemens Healthineers, Enlargen, Germany) by two experienced board-certified nuclear medicine physicians for each centre.

**Figure 1:**
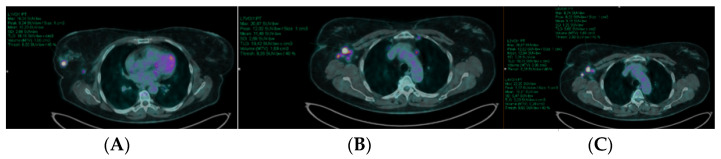
Image analysis is presented in the figure (transaxial fused images of a patient performing baseline [^18^F]FDG PET/CT). Breast primary tumor (**A**) and the most representative axillary lymph node metastasis (**B**) were contoured with a circular ROI. The system automatically adapted the ROI into a 3-d VOI and calculated semiquantitative parameters as shown in the figure and reported in the text. Moreover, all lesions showing increased [^18^F]FDG uptake were contoured (**C**) to obtain the whole body load of disease, in terms of MTV_WB and TLG_WB.

At the visual analysis, pathological findings were considered focal area(s) of increased tracer uptake or diffusely increased uptake, excluding sites of physiological distribution, in comparison with surrounding tissues. Circular regions of interest (ROIs) were drawn around the primary tumor lesion (B), the axillary lymph nodes (N), extra-axillary lymph nodes (DN) and metastases with focally increased uptake in transaxial slices (Figure 1). The system automatically adapted the ROI into 3-dimensional volume of interest (VOI) and calculated the following parameters: standardized uptake value (SUV)max, SUVmean, metabolic tumor volume (MTV)—defined as the tumor volume with at least 40% uptake of the SUVmax within the VOI—and total lesion glycolsis (TLG)—calculated multiplying SUVmean and MTV. Semiquantitative parameters of primary tumor (SUVmax_B; SUVmean_B; MTV_B; TLG_B) and reference lesions (the most representative one in term of uptake intensity and size) for N (SUVmax_N; SUVmean_N; MTV_N; TLG_N) and DN (SUVmax_DN; SUVmean_DN; MTV_DN; TLG_DN) were reported; moreover, the sum of MTV and TLG values ofevery detected lesion in the scanwere calculated(whole body MTV_WB and TLG_WB).

### 2.5. Statistical Analysis

Categorical variables were defined as number (%) and continuous data as median (range) groups or as mean standard deviation. The differences among categorical variables were assessed using the chi-square test, while ANOVA was used to compare the distribution of data among different group of patients. Moreover, Student’s *t* test was used to compare the differences between two independent groups of patients. Receiver operating characteristics (ROC) curves were constructed for continuous variables in order to define the best cut-off values. For each ROC curve, the AUC was calculated. For statistical tests, a *p*-value ≤ 0.05 was considered the threshold for significance. The statistical analysis was performed by using MedCalc software version 20.115—32-bit (MedCalc Software Ltd., licensed by L.E.).

## 3. Results

### 3.1. [^18^F]FDG PET/CT Results

Overall, 133 patients were evaluated (34 from Centre A and 99 from Centre B). Luminal groups were: 9 (6.8%) Luminal A, 49 (36.8%) Luminal B, 33 (24.8%) Luminal B + HER-2, 10 (7.5%) HER-2 enriched and 32 (24.1%) TNBC. Patients with luminal A and HER-2 enriched BC were excluded from the analysis due to their small sample size. Details of patients’ population are reported in Table 1.

Overall, pCR after NAC was achieved in 47 patients (41.2%). Among the different groups, pCR was more frequently reached in Luminal B + HER-2 BC (51.5%) and TNBC (59.4%), while only 22.4% of Luminal B patients were responders (*p* = 0.002).

**Figure 2:**
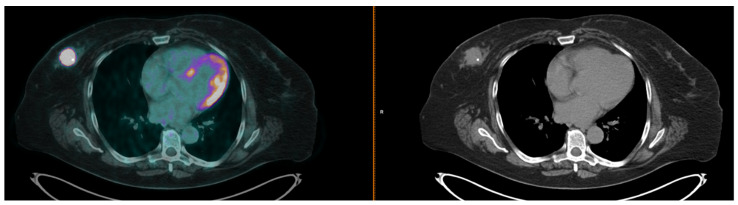
Transaxial fused (on the left) and CT images (on the right) of a baseline [^18^F]FDG PET/CT performed in a patient with TNBC, before starting NAC. A focal area of uptake can be seen in the right breast tumor. On CT images, a clip positioned within the lesion during the biopsy can be observed.

[^18^F]FDG PET/CT detected the primary tumor in almost every patient (98.3%; Figure 2). Moreover, lymph node metastases were more frequently detected in Luminal B patients (65.3% in axillary lymph nodes and 26.5% in distant lymph nodes). [^18^F]FDG PET/CT results are reported in Table 2.

### 3.2. [^18^F]FDG PET/CT Semiquantitative Data and Response to NAC

The analysis of semiquantitative parameters in the 3 subgroups is reported in Table 3. In the Luminal B subgroup a statistically significant difference in median SUVmean_B values was found between responders (7.06 ± 5.9) and non-responders to NAC (4.4 ± 2.1) (*p* = 0.027). Therefore, patients with a higher SUVmean_B value at baseline [^18^F]FDG PET/CT were more likely to achieve pCR after NAC. Conversely, Luminal B + HER-2 non-responders showed a statistically significantly higher median MTV_B (7.3 ± 4.2 cm^3^ vs. 3.5 ± 2.5 cm^3^; *p* = 0.003) and TLG_B (36.5 ± 24.9 vs. 18.9 ± 17.7; *p* = 0.025) than responders at baseline [^18^F]FDG PET/CT. None of the volumetric parameters could predict pCR after NAC in TNBC.

The best cut-offs of volumetric parameters for predicting pCR after NAC were calculated using ROC curves. The statistically significant results are displayed in Table 4. MTV_B and TLG_B could discriminate responders vs. non-responders both in Luminal B (cut-off: ≤3.9 cm^3^ and ≤32.87; AUC = 0.72 and AUC = 0.68, respectively) and Luminal B + HER-2 BC (cut-off: ≤4.78 cm^3^ and ≤25.6; AUC = 0.76 and AUC = 0.75, respectively). Similarly, the cut-off values of MTV_wb (cut-off: ≤17.7 cm^3^; AUC = 0.73) and TLG_wb (cut-off: ≤61.1; AUC = 0.68) could predict pCR after NAC in Luminal B patients. Conversely, no cut-off values for PET volumetric parameters able to predict pCR in patients with TNBC were found.

### 3.3. [^18^F]FDG PET/CT Semiquantitative Data and Survival

**Figure 3:**
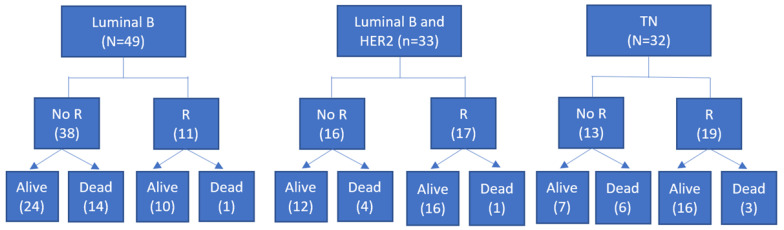
Survival analysis for patients of the different subgroups, stratified for PCR response to NAC. No R = no response after NAC; R = pCR after NAC; TN = triple negative.

The median follow-up for DFS was 73.9 (1–129) months for Luminal B patients, 37.6 (10–129) months for Luminal B + HER-2 patients and 40 (3–125) months for TNBC patients. Nineteen (38.8%), 6 (18.2%) and 10 (31.3%) patients with Luminal B, Luminal B + HER-2 and TNBC, respectively, had a recurrence of disease. The median follow-up for OS was 86 (1–129) months for Luminal B patients, 40 (10–130) months for Luminal B + HER-2 patients and 40 (9–125) months for TNBC patients. Overall, 29 (25.4%) patients died during follow-up. Of those, 15 (51.7%) had Luminal B BC, 5 (17.2%) Luminal B + HER-2 BC, and 9 (31.0%) TNBC. A further survival analysis was performed dividing the patients based on pCR after NAC: responders vs. non-responders. The results are reported in Figure 3.

Interestingly, among Luminal B patients who did not achieve pCR after NAC, median MTV_N, TLG_N, and TLG_WB were higher in patients dead at follow-up (respectively, 10.4 ± 16.6 cm^3^ vs. 52.2 ± 83.6 cm^3^ *p* = 0.05; 46.3 ± 74.4 vs. 296 ± 458 *p* = 0.03 and 227.5 ± 376.7 vs. 674.4 ± 908.7 *p* = 0.05). Similarly, among Luminal B + HER-2 patients who did not reach pCR after NAC those dead at follow-up had higher median SUVmax_N and SUVmean_N (respectively, 3.7 ± 1.0 vs. 16.7 ± 6.1 *p* = 0.03 and 2.6 ± 0.5 vs. 5.9 ± 1.4 *p* = 0.03). Moreover, in patients with TNBC who achieved pCR after NAC, 4 volumetric parameters resulted significantly higher in patients who died during follow-up: MTV_B, TLG_B, MTV_WB and TLG_WB (Table 5).

## 4. Discussion

**Figure 4:**
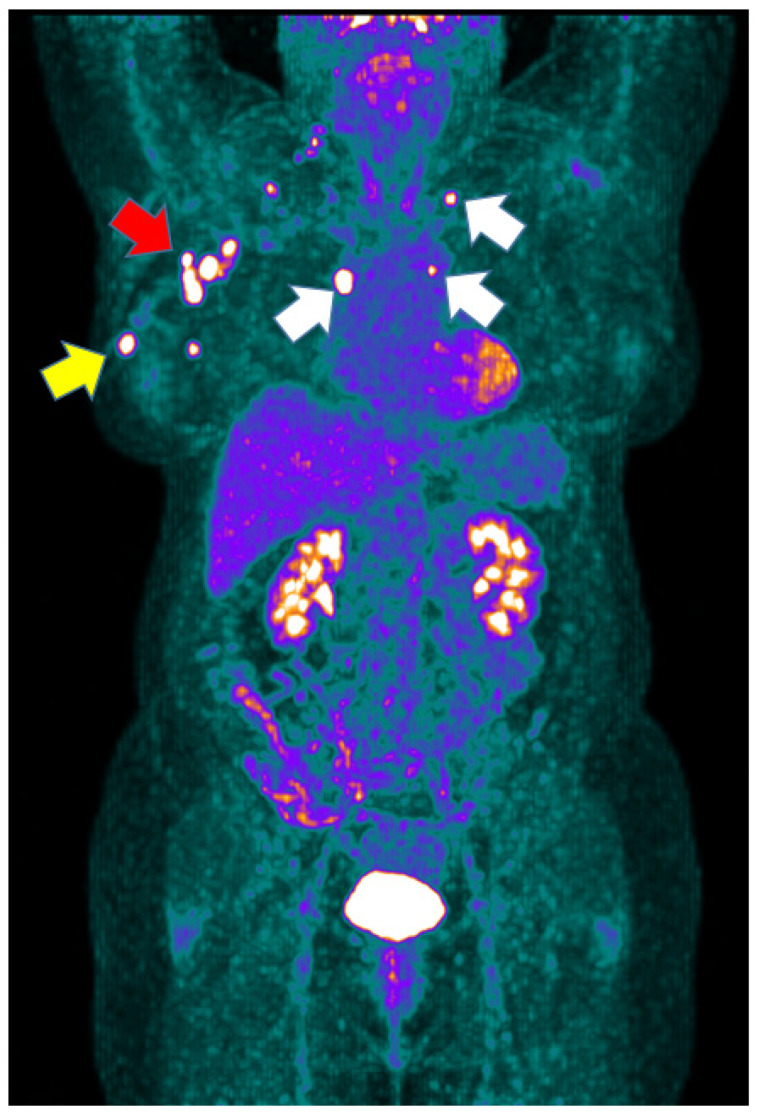
Maximum intensity projection (MIP) of a Luminal B patient performing baseline [^18^F]FDG PET/CT prior to starting NAC. MIP shows multiple areas of increased uptake in correspondence of the primary tumor (yellow arrow) and of multiple axillary (red arrow) and distant (white arrows) lymph node metastases.

BC is usually a tumor over-expressing GLUT 1–3 and, therefore, is evaluable with [^18^F]FDG PET/CT [18]. However, the different subtypes of BC present different [^18^F]FDG avidities, in relation to their diverse glucose metabolism. TNBC and HER-2 enriched BC usually present a high [^18^F]FDG uptake, whereas luminal BC are usually characterized by a faint [^18^F]FDG uptake, in particular luminal A [19]. In our cohort of patients affected by different molecular subtypes of BC, baseline [^18^F]FDG PET/CT detected 98.3% of primary tumors, confirming its accuracy in aggressive BC [20,21]. Moreover, axillary lymph node metastases and distant lymph node metastases were detected in 64 (56.1%) and 26 (22.8%) patients, respectively (Figure 4). Lymph node metastases were more frequently detected in patients affected by Luminal B BC, in keeping with the most updated guidelines that suggest to perform NAC only in high-risk Luminal B BC after a multidisciplinary discussion [15]. Our results support the use of [^18^F]FDG PET/CT for the baseline staging of patients with aggressive BC addressed to NAC. With this aim, MRI might be superior to [^18^F]FDG PET/CT in primary tumor evaluation, but PET/CT provides a whole body disease evaluation. Therefore, the two imaging modalities could play a synergic role in the future for this subset of patients; however, the cost-effectiveness of this proposed strategy needs to be further evaluated.

Semiquantitative parameters extracted from PET/CT data saw a rising use in the last decade, both for [^18^F]FDG and non-[^18^F]FDG imaging [22,23,24]. Among those, SUVmax is the most commonly utilized, but volumetric parameters have recently been growing in terms of diffusion and interest. MTV defines the volume of metabolic active tumor, whereas TLG combines the volume and the intensity of the metabolic activity of a lesion. Moreover, the sum of the values of the volumetric parameters for each [^18^F]FDG uptake could provide a quantification of the whole body tumor burden, which is relevant for patients’ prognosis.

A few papers investigated the correlation between semiquantitative parameters extracted from [^18^F]FDG PET/CT and pCR after NAC. In particular, Galán et al. [25] failed to identify a role of volumetric parameters in pCR prediction. Conversely, Evangelista et al. [26] showed a predictive value of TLG_WB for DFS, and Hyun et al. [27] reported that the variation of MTV_WB was predictive for OS at multivariate analysis in BC patients treated with NAC. However, these studies considered BC patients regardless of the molecular subgroup. In our work, we aimed to identify semiquantitative parameters extracted from baseline [^18^F]FDG PET/CT that could predict pCR after NAC in three different molecular subgroups of patients, being different in terms of NAC regimen, chemosensitivity, and prognosis. Luminal B patients who achieved pCR after NAC had significantly higher median SUVmean_B than non-responders. This means that the metabolic activity of the primary tumor seems to be the main feature for predicting NAC results in Luminal B patients. In Luminal B + HER-2 patients, volumetric parameters of the primary tumor resulted significantly lower in responders vs. non-responders. As a consequence, we can speculate that for this sub-group of patients the main feature to consider is the extension of the primary tumor. Surprisingly, we did not find any semiquantitative parameter able to predict pCR after NAC in TNBC patients. Perhaps PET-derived radiomic features and artificial intelligence could provide better results for this clinical target, even though standardization and time-reducing automated software are still lacking for routine radiomic use [28,29,30,31]. The possibility to predict pCR after NAC from baseline imaging would be of utmost clinical relevance. Indeed, detecting responders could induce a calibration of the treatment regimen (reduced number of schemes and, consequently, treatment-related toxicities and optimized time to surgery), while identifying non-responders could induce the clinicians to select a tailored therapeutic approach and a shorter time revaluation. As a consequence, we tried to calculate the best cut-offs of semiquantitative parameters to help the clinicians to early identify patients who could have a reduced possibility of achieving pCR after NAC. We believe that every centre should calculate their own cut-off values for this purpose, as they are relatively easy to obtain and could provide an added value in clinical daily practice. Hopefully, the widespread of automated software will speed up the extraction of these data from PET/CT images, allowing a wider use of semiquantitative parameters in future daily clinical practice.

Several papers have already correlated [^18^F]FDG PET/CT results and prognosis in patients affected by different molecular subtypes of BC and in several settings of disease [20,32,33]. As expected we found that several median baseline volumetric parameters were higher in patients non-responders to NAC than the counterpart and also in died patients than those who alived, both for Luminal B (MTV_N, TLG_N and TLG_wb) and Luminal B + HER-2 patients (SUVmax_N, SUVmean_N). This is consistent with the multiple literature evidence reporting a strong negative prognostic factor of residual disease burden in BC, which is more likely to remain in patients with a higher baseline burden of disease [8,34]. However, we also found that among TNBC patients who achieved pCR after NAC, the median values of 4 volumetric parameters were significantly higher in patients dead vs. alive at follow-up. Interestingly these parameters are all volumetric parameters, referring to the primary tumor (MTV_B and TLG_B) and to the whole body burden of disease (MTV_wb and TLG_wb). To the best of our knowledge, this is the first paper reporting the potential of [^18^F]FDG PET/CT in predicting the outcomes of TNBC patients achieving pCR after NAC. Moreover, this result was found for TNBC, which is the most aggressive molecular subtype of BC [35]. We believe that this result could have a very high relevance in daily clinical practice if confirmed in larger sample trials.

Finally, this study is not devoid of limitations. Main limitations are the retrospective design of the study and the lack of sample size calculation. A second mild limitation derives from the two similar, but still different, last generation PET tomographs of the same company (Siemens Medical Solutions) in the two hospitals; although we adopted the same acquisition protocol according to the EANM guidelines [17], no phantom-based harmonization strategy was tested to reduce interscanner quantification variability. Moreover, the number of patients enrolled was inhomogeneous between the 2 centres (34 vs. 99) due to the different catchment area. A limit in the survival analysis derives from the low mortality rate of our patient population (low number of events at follow-up) and the small size of the molecular subgroups.

## 5. Conclusions

This work confirmed literature data reporting the high accuracy of baseline [^18^F]FDG PET/CT for the systemic staging in BC patients. [^18^F]FDG PET/CT could carve out a synergic role together with other imaging tools for a more accurate evaluation of these patients at diagnosis.

The analysis of semiquantitative parameters demonstrated that the metabolic activity of the primary tumor (SUVmean_B) and the extension of the primary tumor (MTV_B and TLG_B) seem to be the main features to consider for predicting pCR after NAC in Luminal B and Luminal B + HER-2 patients, respectively. Semiquantitative parameters were unreliable for predicting responders vs. non responders to NAC in TNBC; [^18^F]FDG PET-derived radiomic features could be tested for this purpose. Finally, 4 volumetric parameters derived from [^18^F]FDG PET/CT could predict the outcomes in terms of survival of TNBC patients achieving pCR after NAC. If confirmed in further studies this result could open up to a larger use of [^18^F]FDG PET/CT as a baseline prognostic tool in TNBC.

## Figures and Tables

**Table 1:** Characteristics of patient populations. The table displays only patients with Luminal B, Luminal B + HER-2 and TNBC.

Variables	Luminal B	Luminal B + HER-2	TNBC
N	49	33	32
Median age (range), years	50 (30–73)	48 (27–77)	51 (32–73)
Clinical stage I II IIIA IIIB IV NA	4 (8.2%) 11 (22.4%) 10 (20.4%) 12 (24.5%) 9 (18.4%) 3 (6.1%)	5 (15.2%) 9 (27.3%) 12 (36.4%) 2 (6.1%) 1 (3%) 4 (12.1%)	8 (25%) 9 (28.1%) 9 (28.1%) 1 (3.1%) 3 (9.4%) 2 (6.3%)
Histology ILC IDC Mixed NA	7 (14.3%) 41 (83.7%) 1 (2%) 0	7 (21.2%) 25 (75.8%) 0 1 (3%)	4 (12.5%) 28 (87.5%) 0 0
Grade G1 G2 G3 Unknown	1 (2%) 12 (24.5%) 34 (69.4%) 2 (4.1%)	1 (3%) 5 (15.2%) 22 (66.7%) 5 (15.2%)	0 2 (6.3%) 27 (84.4%) 3 (9.4%)
ER expression No Yes	5 (10.2%) 44 (89.8%)	9 (27.3%) 24 (72.7%)	32 (100%) 0
PR expression No Yes	13 (26.5%) 36 (73.5%)	11 (33.3%) 22 (66.7%)	32 (100%) 0
Ki67 median (range), %	35 (14–90)	38 (10–80)	63 (5–90)
HER-2 expression No Yes	49 (100%) 0	0 33 (100%)	32 (100%) 0
Trastuzumab No Yes	49 (100%) 0	0 33 (100%)	32 (100%) 0
pCR after NAC No Yes	38 (77.6%) 11 (22.4%)	16 (48.5%) 17 (51.5%)	13 (40.6%) 19 (59.4%)

IDC = invasive ductal cancer; ILC = invasive lobular cancer; NA = not available.

**Table 2:** Results of [^18^F]FDG PET/CT in the 3 subgroups of patients.

	Luminal B (n = 49)	Luminal B + HER-2 (n = 33)	TNBC (n = 32)	*p* Value
Breast PET No Yes	0 49 (100%)	1 (3%) 32 (97%)	1 (3.1%) 31 (96.9%)	0.464
Axillary LN PET No Yes	17 (34.7%) 32 (65.3%)	18 (54.5%) 15 (45.5%)	15 (46.9%) 17 (53.1%)	0.190
Distant LN PET No Yes	36 (75.3%) 13 (26.5%)	26 (78.8%) 7 (21.2%)	26 (81.3%) 6 (18.8%)	0.693

LN = lymph nodes.

**Table 3:** Analysis of semiquantitative parameters for predicting pCR after NAC in the 3 subgroups.

	Luminal B (n = 49)			Luminal B + HER-2 (n = 33)			TNBC (n = 32)		
	No Response to NAC	Response to NAC	*p* Value	No Response to NAC	Response to NAC	*p* Value	No Response to NAC	Response to NAC	*p* Value
SUVmax_B	10.3 ± 6.4	11.1 ± 9.2	0.732	9.3 ± 5.1	9.5 ± 4.9	0.917	17.6 ± 12.1	14.9 ± 7.7	0.443
SUVmean_B	4.4 ± 2.1	7.06 ± 5.9	0.027	4.9 ± 2.1	5.1 ± 2.4	0.808	7.1 ± 3.9	10.5 ± 9.9	0.240
MTV_B	67.9 ± 135	8.8 ± 12.9	0.160	7.3 ± 4.2	3.5 ± 2.5	0.003	38.1 ± 63	11.9 ± 17.9	0.095
TLG_B	296.4 ± 584	115.8 ± 292.9	0.330	36.5 ± 24.9	18.9 ± 17.7	0.025	447.7 ± 1055	121.7 ± 182.3	0.194
SUVmax_N	10.4 ± 8.3	10.3 ± 9.9	0.977	8.9 ± 7.8	12.2 ± 8.1	0.478	4.9 ± 3.5	9.1 ± 4.8	0.063
SUVmean_N	4.1 ± 1.8	6.3 ± 5.9	0.105	3.9 ± 1.9	6.7 ± 4.5	0.222	3.2 ± 1.8	3.9 ± 1.9	0.403
MTV_N	22.6 ± 48.9	3.3 ± 4.6	0.278	1.5 ± 1.4	37.3 ± 100.9	0.451	1.4 ± 1.7	11.8 ± 14.9	0.055
TLG_N	119.2 ± 268.3	23.1 ± 47.1	0.327	7.9 ± 10.6	31.1 ± 31.2	0.135	4.3 ± 4.7	51 ± 70.6	0.065
SUVmax_DN	11.5 ± 8.4	5.9 ± 2.5	0.289	5.7 ± 1.01	12.9 ± 11	0.317	3.4 ± 1.4	5.9 ± 2.8	0.314
SUVmean_DN	5.1 ± 2.9	3.9 ± 1.9	0.518	3.8 ± 0.4	6.9 ± 6.2	0.416	2.3 ± 0.4	3.5 ± 1.5	0.338
MTV_DN	5.9 ± 5.7	0.7 ± 0.4	0.150	1.1 ± 0.7	1.4 ± 1.2	0.691	0.7 ± 0.2	2.2 ± 2.3	0.436
TLG_DN	40 ± 45.6	2.13 ± 1.1	0.327	3.9 ± 2.3	9.2 ± 7.1	0.281	1.6 ± 0.6	6.7 ± 5.6	0.293
MTV_WB	83.8 ± 140.9	11.3 ± 10.3	0.097	33.6 ± 101.3	8.2 ± 7.1	0.310	39.1 ± 64.3	17.6 ± 20.1	0.180
TLG_WB	382.3 ± 648.5	90.4 ± 141.3	0.148	39.6 ± 26.7	50.1 ± 48.6	0.449	450.9 ± 1057.4	145.4 ± 180.7	0.224

B = breast, N = lymph node, DN = distant lymph nodes, WB = whole body. MTV values are measured in cm^3^.

**Table 4:** Best cut-off values for [^18^F]FDG PET-derived volumetric parameters for predicting pCR after NAC in Luminal B and Luminal B + HER-2 patients.

	Luminal B (n = 49)					Luminal B + HER-2 (n = 33)				
	AUC	Cut-off	Sens	Spec	*p* Value	AUC	Cut-off	Sens	Spec	*p* Value
MTV_B	0.72	≤3.9	63.64%	84.21%	0.01	0.76	≤4.78	88.24%	68.75%	0.002
TLG_B	0.68	≤32.87	72.73%	65.79%	0.03	0.75	≤25.6	88.24%	75%	0.009
MTV_WB	0.73	≤17.7	81.82%	60.53%	0.002	-	-	-	-	-
TLG_WB	0.68	≤61.1	81.82%	65.79%	0.03	-	-	-	-	-

AUC = area under the curve, B = breast, Sens = sensitivity, Spec = specificity, WB = whole body. MTV values are measured in cm^3^.

**Table 5:** Survival analysis of volumetric parameters in TNBC patients who reached pCR after NAC.

	TNBC with pCR after NAC		
	Alive	Dead	*P* Value
MTV_B	7.5 ± 9.9	35.2 ± 34.1	0.009
TLG_B	85 ± 125	317 ± 355	0.039
MTV_WB	12.8 ± 15.2	43.2 ± 26.7	0.012
TLG_WB	105.1 ± 131.4	360.1 ± 286.4	0.020

MTV values are measured in cm^3^.

## Data Availability

Datasets available upon request.
